# Synchronous primary tumors amd distant metastasis detected on ^18^F-FDG PET in patients with head and neck carcinoma

**DOI:** 10.22038/aojnmb.2024.76461.1536

**Published:** 2025

**Authors:** Nitin Gupta, Samta Kumari, Poorva Vias, Manpreet Kaur, Shalini Verma

**Affiliations:** 1 Department of Nuclear medicine, Dr Rajendra Prasad Government medical college,Tanda Kangra Himachal Pradesh, India; 2 Department of Nuclear medicine, Shree Balaji superspeciality hospital, Kangra Himachal Pradesh, India

**Keywords:** Head and neck cancer, Distant metastases, Synchronous tumor, PET/CT, ^18^F-FDG

## Abstract

**Objective(s):**

^18^F-FDG PET/CT has been used to characterize the primary lesion and staging in head and neck cancers (HNC). However, prior studies for detecting distant metastasis and synchronous tumors are sparse, especially in Indian context. To investigate the frequency and distribution of head and neck carcinomas, distant metastases and synchronous malignancies detected in HNC in a north Indian population.

**Methods:**

Medical records and whole body ^18^F-FDG PET/CT examinations performed for initial staging on a total of 281 newly diagnosed HNC patients between 01/2019 to 31/6/2023 in North India were retrospectively analyzed and reviewed to look for distant metastasis and synchronous tumors.

**Results:**

On whole body ^18^F-FDG PET/CT, distant metastases were detected in 33 (11.7%) patients, all with locally advanced primary tumors corresponding to T category 3 and 4. Lung (6%) and bone (~6.7%) were the most common sites of distant metastasis. In nine patients metastases were detected below the diaphragm. Synchronous malignancies were discovered and histopathologically proven in 22 (7%) patients. Lung carcinoma was the most common synchronous tumor, detected in 9 patients. In seven patients synchronous tumour was detected outside the aerodigestive tract, of which four were below the diaphragm.

**Conclusions:**

Of the distant metastasis diagnosed in 11.7% of HNC patients with TNM tumor category T3 and T4, 3% of metastasis lesions were detected below the diaphragm. Synchronous malignancy was diagnosed in 7% patients irrespective of primary HNC stage. These findings demonstrate the advantage of using whole body ^18^F-FDG PET/CT as an ideal and preferred modality for initial staging and screening of HNC patients since detection of distant metastasis or a synchronous malignancy changes the management approach in these patients.

## Introduction

 Head and neck cancer (HNC) is the sixth most common malignancy worldwide, with around 800,000 new cases and 320,000 deaths annually (1). These malignant tumors include cancers of the oral cavity, oropharynx, hypopharynx, and larynx and are squamous cell carcinomas in 90% of the cases (1). In India, HNC accounted for 30% of all cancers in males and 11 to 16% in females, of all sites of cancer (2).

 The frequency of distant metastases in patients with HNC has been reported, in therange of 4%-25%, with the lungs, bones, and liver being the most frequent reported sites (3-6).The 5-year survival rate does not exceed 80% for patients with localized disease whereas it decreases to 50% in case of regional lymph node involvement, and to 20% when distant metastasis are present at diagnosis (7).

 By definition, synchronous primary tumor is defined as a tumor occurring simultaneously or within 6 months of diagnosis of primary tumor (8). Distant metastasis usually occurs late during the course of the disease whereas synchronous malignancies may present at any stage. It is estimated that synchronous tumors are present in 1% to 6% of patients with newly diagnosed HNC (9, 10). 

^18^F-FDG PET/ CT scan is being increasingly utilized for initial staging, detection of recurrent tumors and for localization of primary tumour in cases of HNC and metastasis from unknown origin. Some studies have shown that ^18^F-FDG PET/CT is superior to conventional work up to assess remote metastasis and to detect occult synchronous primary tumours (3, 11). 

 However, pre-treatment ^18^F-FDG PET/CT scan is currently recommended only for detecting metastatic pathology in locally advanced cases of HNC (12). Change in staging and impact on clinical management of patients after integrating ^18^F-FDG PET/CT as part of the initial work up remains poorly understood and not clearly reported in literature.

 Therefore we undertook this retrospective study with the objective to find out frequency of detection and pattern of distant metastasis and occurrence of synchronous tumours in patients with HNC with ^18^F-FDG PET/CT scans as in such a scenario ^18^F-FDG PET/CT may provide additional information and improve HNC staging regardless of clinical disease stage, thus leading to a potential change in patient management.

## Methods

***Patient population***

 In this retrospective study, all patients diagnosed with a primary HNC who underwent a whole body ^18^F-DG PET/CT at the department of PET/CT and nuclear medicine between January 2019 and June 2023 were included. All patients had underwent clinical examination, panendoscopy, head and neck CT, and/or MRI depending on primary tumor location within 4 weeks after diagnosis.

 Exclusion criteria were previous history of head and neck cancer diagnosis or treatment started more than 6 months before ^18^F-FDG PET/CT scan and cervical lymph node metastases of unknown primary tumor.

***PET/CT protocol***

 Patients underwent scans on a General Electric DISCOVERY STE 16 slice PET/CT scanner (GE Health Systems, Milwaukee, WI) after 6 hours of fasting. Imaging was done 1 hour after intravenous administration of approximately 300 to 370 MBq of ^18^F-FDG. A standard acquisition protocol and reconstruction was applied for whole body PET/CT scans. A regional PET/CT acquisition was made separately for the head and neck region with high resolution reconstruction using a slice thickness of 1.2 mm in the head and neck area. Total scanning time was 30 min. The CT scans and corresponding PET data were fused for integrated interpretation.

***Data collection***

 FDG PET/CT reports and images were collected from departmental case record files and PACS system. Sex, age, TNM classification, histopathology, and synchronous malignancies were recorded. Primary tumors were divided into seven different tumor sites according to the Union for International Cancer Control (13), i.e. nasal cavity and sinuses, nasopharynx, oral cavity, oropharynx, hypopharynx, larynx, and salivary glands. Histopathological diagnosis of HNC was noted for each case. 

 Criteria originally described by Warren and Gates (14) were used to identify and define the second primary cancer as follows: 

1) Each tumor must be geographically separate and distinct (it is considered as multicentric primary if the intervening mucosa shows dysplasia). The second primary had to be separated from the first primary by at least 2 cm of normal epithelium. 

2) The possibility that the second primary representing the metastasis or relapse must be excluded.

 3) The index tumor and the second primary tumors should be histologically confirmed.

 In case of lung lesions where biopsy could not be done or was not available, the lesion was interpreted as synchronous second primary, if the lesion was spiculated solitary FDG positive mass and as metastasis when few to multiple peripheral non spiculated or smooth walled and well demarcated solid nodules were present.

***Statistical analysis***

 Nominal and categorical variables were presented as frequencies and percentages. 

 Parametric distributed data were presented as mean±standard deviation. Statistical analyses were performed using MedCalc for Windows, version 19.4 (MedCalc Software, Ostend, Belgium). Sensitivity, specificity, positive predictive value, negative predictive value, and accuracy of ^18^F-FDG PET/CT for synchronous primary tumor were calculated.

## Results

 A total of 281 patients were included in the final study. Of these 205 (73%) were male and 76 (27%) were females. Mean age of the patient was 57 years (age range 34–86 years) (Table 1).

**Table 1:** Showing the demographics and distribution of synchronous tumors and distant metastasis among head and neck cancer patients

	Number of patients	Patients with synchronous tumor	Patients with distant metastasis
SEX	**N (%)**	**N (%)**	**N (%)**
**Male**	205 (~73%)	15 (~7.3%)	27 (~13%)
**Female**	76 (~27%)	7 (~9%)	6 (~8%)
AGE GROUP			
**30-50 years**	89 (~31%)	4 (~4.4%)	9 (10%)
**51 – 70 years**	114 (~40%)	13 (~11.4%)	18 (~15%)
**71-90 years**	78 (~27%)	5 (~6.4%)	6 (7.6%)

***Localization and histopathological types of primary tumour***

 Oral cavity carcinoma was found in 125/281 (44%) patients, followed by hypopharynx, in 57(~20%) patients (Table 2). The most common histopathological type of HNC was squamous cell carcinoma (SCC) found in 238(~84%) patients, followed by adeno-carcinoma in 12 (4%), lymphoma in 9 (3%), poorly differentiated carcinoma in 6 (2%) patients, adenoid cystic carcinoma in 3 patients whereas sarcoma was found in 2 patients while neuroendocrine and desmoplastic tumors were found in one patient each.

**Table 2:** Location of the primary tumor and number of patients with synchronous tumors and distant metastasis in relation to specific subsites of head and neck cancer

Site of Primary malignancy	Number of Patients N (%)	Number of patients with synchronous primary (N)	Number of patients with distant metastasis (N)
Oral cavity	125 (44%)	8	12
Oropharynx	38(13%)	2	7
Hypopharynx	57(20%)	7	3
Nasopharynx	19(6%)	2	3
Larynx	24(8%)	1	1
Salivary glands	8(2%)	2	4
Maxilla	5(2%)	0	1
Mandible	1	0	1
Nasal cavity & PNS	4(1%)	0	1

***Detection and Localization of distant metastases***

 On ^18^F-FDG PET/CT scans, distant metastases were found in 33/281 (~11.7%) patients with primary HNC patients. Distant metastasis was more commonly detected among male patients (n=27; ~13%) and among age group of 51-70 years (n=18~15%) (Table 1). Lung metastasis was detected and histopathologically proven in eighteen (~6%) patients, skeletal metastasis in nineteen (~6.7%) patients, liver metastasis in six (2%) patients, and adrenal gland metastasis in one patient. In 9 (~3%) patients metastases were detected below the diaphragm. The locations of metastatic lesions are shown in Table 3. In terms of relationship of locoregional staging of the primary tumors to occurrence of distant metastasis, in 21 patients the primary tumors were in T4 and in 12 patients the primary tumor was in T3 category.

**Table 3:** Location of the primary tumor and number of patients with site of distant metastasis in relation to specific subsites of head and neck cancer

**Site of primary tumour **	** Histopathology**	** Stage of primary tumor**	** Site of metastasis**
Anterior Tongue	Squamous cell carcinoma	T3N2b	Lung and mediastinal nodes
Hypopharynx	Squamous cell carcinoma	T3N2b	Supraclavicular lymph nodes, cervical vertebrae
Parotid gland	Adenocarcinoma	T4aN2b	Lung and mediastinal lymph nodes
Larynx	Squamous cell carcinoma	T4a N2c	Lung , supraclavicular and mediastinal lymph nodes
Soft palate	Squamous cell carcinoma	T4a N2b	Lung and pleural metastasis
Buccal mucoca	Squamous cell carcinoma	T4aN2b	Lung nodules, dorso lumbar and pelvic bone metastasis
Nasopharynx	Squamous cell carcinoma	T4aN2c	Supraclavicular nodes, liver, dorsal vertebrae
Base of tongue	Squamous cell carcinoma	T3N2c	Lung and mediastinal nodes
Submandibular gland	Adenoid cystic carcinoma	T4aN2b	Liver, lungs, vertebrae and pelvic bones
Tonsil	Squamous cell carcinoma	T4aN2c	lung , mediastinal nodes and Liver
Maxillary alveolus	Squamous cell carcinoma	T4aN2b	cervical vertebrae, bilateral supraclavicular lymph nodes
Nasopharynx	Squamous cell carcinoma	T4aN2c	Cervico dorsal Vertebral and lung
Buccal mucosa	Squamous cell carcinoma	T4aN2b	Dorsal vertebrae
Parotid gland	Adenoid cystic carcinoma	T3N2b	Lung, mediastinal and anterior diaphragmatic lymph nodes
Gingivobuccal suclus	Squamous cell carcinoma	T4aN2b	Ribs and dorsal vertebrae
Hypopharynx	Squamous cell carcinoma	T4aN2c	Lung and pleura
Mandible	Clear cell odontogenic carcinoma	T4aN2c	Supraclavicular lymph nodes, lung nodules. Dorso- lumar vertebral and pelvic metastasis
Lateral Tongue	Squamous cell carcinoma	T4aN2c	Supraclavicular and axillary lymph nodes
Retromolar trigone	Squamous cell carcinoma	T3N2b	Dorso lumbar Vertebral and pelvic bones
Base of tongue	Basaloid Squamous cell carcinoma	T3 N2b	Lung
Floor of mouth	Squamous cell carcinoma	T3N2b	Supraclavicular lymph nodes, liver
Buccal mucoca	Squamous cell carcinoma	T4aN2c	Supra and infra clavicular , axillary and mediastinal lymph nodes, dorsal vertebral lung and liver metastasis
Maxillary sinus	Squamous cell carcinoma	T4aN2b	Cervico dorsal Vertebral and manubrial metastasis
Tonsil	Squamous cell carcinoma	T3N2b	lung and mediastinum
Hard palate	Squamous cell carcinoma	T3N2b	Lung , pleura, liver, adrenal
Nasopharynx	Squamous cell carcinoma	T3N3b	Supra and Infra clavicular lymph nodes, Dorsal vertebrae
Buccal mucosa	Squamous cell carcinoma	T3N2b	Manubrium, dorsal vertebrae
Hypopharynx	Squamous cell carcinoma	T3N2b	Dorso lumbar Vertebra, and lung nodules
Gingivobuccal sulus	Squamous cell carcinoma	T4aN2c	Cervical vertebrae, scapular and ribs metastasis
Retromandibular trigone	Squamous cell carcinoma	T4bN2b	Cervical and dorsal vertebral metastasis
Tonsil	Squamous cell carcinoma	T4aN2b	Supraclavicular, infra clavicular and mediastinal nodes
Submandibular gland	Adenoid cystic carcinoma.	T4aN2c	Dorsal Vertebral and rib metastasis
Base of tongue	Squamous cell carcinoma	T4aN2c	Mediastinal lymph nodes, lung nodules, dorso lumbar vertebra and pelvic skeletal metastasis

***Detection and localization of Synchronous tumours***

 In 27 patients ^18^F-FDG PET/CT was suggestive of a synchronous tumor. Of these in 22 patients, a synchronous tumor was histopathologically confirmed, where as in 5 patients inflammatory/ infective pathology was found. Of the 22 patients with hitopathologically proven synchronous tumor, lung cancer was detected in 9 cases while in 6patients synchronous tumor was detected in upper aero digestive tract, and in 7patients synchronous tumor was detected outside the aerodigestive tract, of which 4 synchronous tumors were found below the diaphragm. The distribution of various synchronous malignancies is shown in Table 4. The sensitivity, specificity, positive predictive value, negative predictive value and accuracy of ^18^F-FDG PET/CT for identification of synchronous primary was found to be 100.0% (95% C.I~ 84.5% to 100%) , 98% (95% C.I.~ 95.5% to 99.3%), 81.8% (95% C.I~ 65.41% to 91.5%), 100% (95% C.I~ 98.5% to 100)% and 98.% (95% C.I 95.9% to 99.4%) respectively.

**Table 4:** Location of the primary tumor and number of patients with synchronous tumors in relation to specific subsites of head and neck cancer

**Site of primary tumour**	**H** **istopathology**	**Stage of ** **primary tumor**	**Site of synchronous tumor**
Hypopharynx	Squamous cell carcinoma	T2N1	Tonsil
Floor of mouth	Squamous cell carcinoma	T2N2b	Tongue
Buccal mucosa	Squamous cell carcinoma	T1N2b	Breast
submandibular gland	Squamous cell carcinoma	T1 N2a	Thyroid
Hypopharynx	Squamous cell carcinoma	T2 N2b	Lung
Hypopharynx	Squamous cell carcinoma	T2N2a	Esophagus
Retromolar trigone	Squamous cell carcinoma	T2N2b	Endometrium
Hypopharynx	poorly differentiated carcinoma	T3N2b	Pancreas
Base of tongue	Squamous cell carcinoma	T1N2b	lung
Hypopharynx	Squamous cell carcinoma	T2N0	Floor of mouth
Nasopharynx	Squamous cell carcinoma	T1N1	Prostate
Gingivobuccal sulcus	Squamous cell carcinoma	T2N2b	Thyroid
Parotid gland	Adenoid cystic carcinoma	T2N2b	Lung
Hypopharynx	Squamous cell carcinoma	T3N2b	Stomach
Retromolar trigone	Squamous cell carcinoma	T3N2b	Lung
Base of tongue	Basaloid SCC	T4a N2c	Lung
Floor of mouth	Squamous cell carcinoma	T2N2b	Esophagus
Tongue	Squamous cell carcinoma	T3N2b	Retromolar trigone
Nasopharynx	Squamous cell carcinoma	T2N2a	Lung
Larynx	Squamous cell carcinoma	T2N2b	Lung
Buccal mucosa	Squamous cell carcinoma	T1N2a	Lung
Hypopharynx	Squamous cell carcinoma	T3N2b	Lung

## Discussion

 In the present study, most common site of the primary or index case was oral cavity (44%) followed by hypopharynx (20%) (Table 2).The mean age of the patients with primary HNC tumors was 57 years. HNC were commoner in males than females with the male:female ratio of 2.7:1. SCC was the most common (84%) histotopathological type of HNC in this study. 

 These finding are similar to finding of study by Siddiqui et al (15) wherein male to female patient’s ratio was 3.1:1, and most common histopathological type was SCC (96%). However in their study the most common reported site for HNC was larynx.

 On staging FDG PET/CT scans, a major number of cases i.e., 191 (68%) were found to be in an advanced stage at the time of referral, corresponding to TNM stage 3 or 4. This finding is in accordance with results and data of some of the studies in India where 60 to 80% patients presented at TNM stage 3 or 4 (16, 17). 

 On ^18^F-FDG PET/CT scans, 27 patients had lesions suggestive of a synchronous malignancy. 

 In 22 (~7%) patients synchronous primaries were histopathologically proven. The most common age group with synchronous malignancy was between 51-70 years (Table 1). 

 Among other 5 patients, FDG PET/CT proved to be false positive with histopathology revealing a benign /inflammatory pathology. 

 The sensitivity, specificity, positive predictive value, negative predictive value and accuracy of ^18^F-FDG PET/CT for identification of synchronous primary was found to be 100.0%, 98%,81.8%, 100% and 98% respectively. In study by Linz et al (18), ^18^F-FDG PET/CT detected all malignancies within the whole body (sensitivity: 100%) and yielded false-positive results in four cases (specificity: 97.6%); Sensitivity of ^18^F-FDG PET/CT: 100% vs panendoscopy: 87.5%), specificity (99.4% vs 100%), negative predictive value (100% vs 99.4%), and positive predictive value (88.9% vs 100%) for detecting synchronous upper aero digestive tract malignant tumor. 

 Strobel et al (19) reported 8% prevalence of synchronous primaries on ^18^F-FDG PET/CT scan at initial staging of head and neck tumors. 

 In a prospective study, Lee et al (20) reported synchronous second primary in the head and neck cancers in 8 patients (2.5%) and found the sensitivity and specificity of ^18^F-FDG PET/CT for identification of second primaries to be 75.0% and 98.7%, respectively. In another retrospective study by Gandla et al (21), synchronous primary tumors were reported in 7 patients (1.49%) and 33 patients (7.03%) were diagnosed with distant metastasis on ^18^F-FDG PET/CT imaging at the time of initial presentation. 

 In the present study, carcinoma of hypo-pharynx was the most common primary tumor associated with synchronous tumor, followed by oral cavity. This finding is in accordance to study by Gandla et al (21) where hypopharynx was the most common primary head and neck cancer associated with synchronous second primary site.

 In our retrospective analysis, 9/22 (40%) of synchronous malignancies were detected in the lung. In study by Strobel et al (19), 46% of the synchronous malignancies in HNC patients had involved the lungs. This association of synchronous lung carcinoma can be attributed to smoking in these patients. Though carcinoma of the lung is usually seen as a metachronous tumour in patients with head and neck cancers (22), it may also present as synchronous primary tomor. Other synchronous tumors were detected in the upper aero digestive tract in 6 and non-aero digestive sites in 7 patients (table 4). Esophageal carcinoma was detected in 2(1%) patients and involved the cervical and upper thoracic esophagus. This finding is in accordance with some of earlier reports of 1-1.83% synchronous malignancy of the esophagus (3). Interestingly in 4 patients, synchronous tumor was detected below the diaphragm (Table 4). In a study by Leclere et al (23), FDG PET/CT detected synchronous tumours in 60 out of 477 HNC patients, synchronous primary were located in the lung, head and neck, esophagus, colon, prostate, liver, breast and stomach in 35.5, 19.4, 17.7, 11.3, 8.1, 3.2, 1.6, and 1.6% of cases respectively.

 To emphasize the utility for whole body FDG PET/CT scan as a valuable screening tool for detecting synchronous malignances, we have described a few interesting representative cases below herein. 

**Figure 1:**
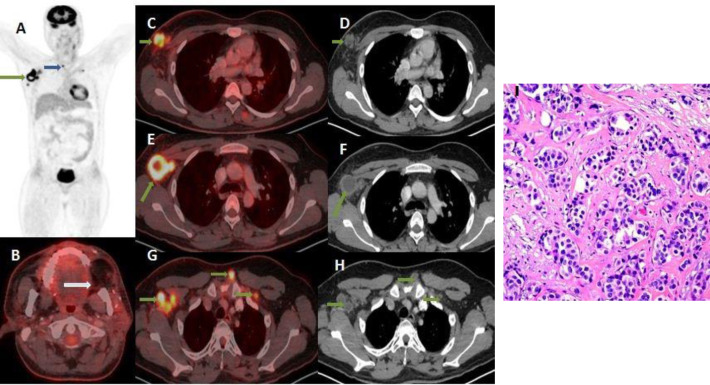
A 63 year old female with carcinoma left side buccal mucosa, post WLE and CRT, 4 months after the initial diagnosis of buccal carcinoma, the patient now presented with complaint of right axillary swelling. PET image (**A**) shows focally increased FDG uptake in the right breast and axillary region (**green arrow**) and in superior mediastinal region (**blue arrow**). Axial fused PET/CT images (**B**) shows no abnormal FDG avid lesion in post WLE left buccal region (**white arrow**). Axial fused PET/CT (**C**) corresponding CT (**D**) images show FDG avid soft tissue lesion in the right breast (**green arrow**). Axial fused PET/CT (**E** and **G**) and corresponding CT images (**F** and **H**) show an enlarged necrotic right axillary lymph node (oblique green arrow) and few other right axillary, station VII and left subpectoral lymph nodes (**all shown with horizontal green arrows**). ** I**) Biopsy of the right breast lesion revealed cells with mild to moderate pleomorphism, with small punctate nuclei, vesicular chromatin, and prominent nucleoli. Note is made of glandular differentiation with tubule formation (H&E; 200X). Final diagnosis of metastatic breast carcinoma (IDC) was made

 A 63 year old female with post WLE and CRT buccal mucosa carcinoma, presented within 4 months of her treament with complaint of right axillary swelling. FNAC was suggestive of metastatic adenocarcinoma. Whole body FDG PET/ CT (Figure 1) showed no abnormal FDG avid lesion in post WLE left buccal region. 

 However, high grade FGD avid enlarged necrotic right axillary (SUV_max_=12.8), station VII and left subpectoral lymph nodes and FDG avid nodular lesion in the right breast (SUV_max_= 10.6). Biopsy of the breast lesion revealed adenocarcinoma.

**Figure 2:**
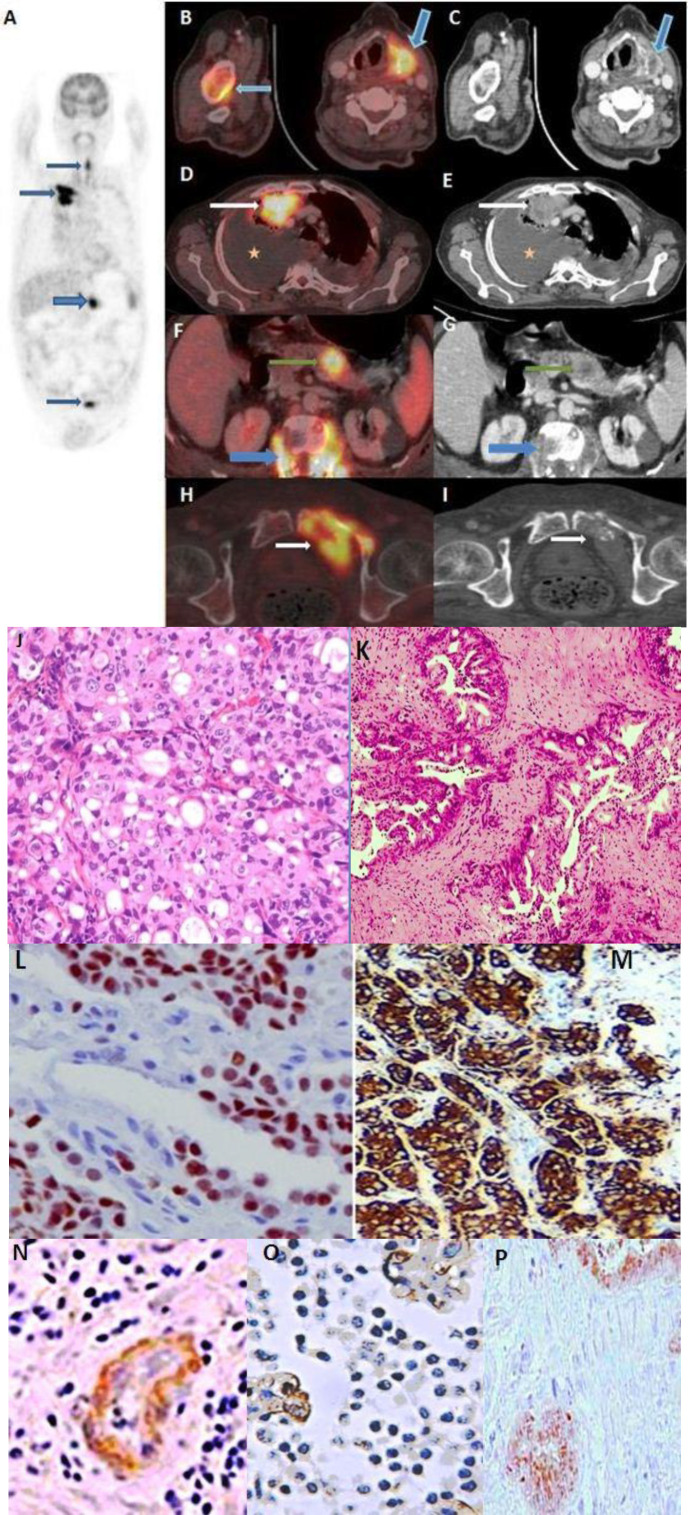
A 49 year old male presented with carcinoma left pyriform sinus and pathological fracture of vertebra with no other history of treatment. PET maximal intensity projection (MIP) image (**A**) shows focally increased FDG uptake in the left side of neck, right thoracic region, in abdominal slightly to left of midline, and in the pelvic region. Axial fused PET/CT images (**B**) and corresponding CT images (**C**) shows FDG avid lesion in left pyriform sinus (**oblique blue arrow**) and along right humeral shaft ( **blue arrow**). Axial fused PET/CT (**D** and **F**) corresponding CT (**E** and **G**) images show FDG avid heterogenous soft tissue lesion in the right lung apex (**white arrow**), FDG avid lytic vertebral lesion with paravertebral soft tissue (**blue arrow**) and FDG avid heterogenous hypodense soft tissue lesion in the pancreatic body (**green arrow**). Axial fused PET/CT (**H**) and corresponding CT images (**I**) show an FDG avid lytic lesion of left pubic ramus (**white arrow**). J) Biopsy of the lung lesion showed tumor composed of solid sheets of intracellular mucin containing vacuolated Cells (H&E: 200X). **K**) Biopsy and histopathology from the pancreatic lesion revealed interconnecting complex glands embedded in desmoplastic stroma. IHC from the lung lesion (**L** and **M**) was, while IHC from the pancreatic lesion (**N**, **O**, **P**) was positive for Pentraxin 3, Maspin and S100P and negative for TTF-1 and Napsin A. Based on biopsy and IHC findings, a diagnosis of synchronous adenocarcinoma of right lung with synchronous pancreatic ductal adenocarcinoma with skeletal metastasis was made

 In an another case (Figure 2) a 49 year old male with known primary hypopharyngeal carcinoma complaining of chest pain and back pain was referred for whole body FDG/CT scan for initial staging. In addition to FDG positive hypopharygeal lesion, high grade FDG avid right lung mass (SUV_max_=13.6), FDG avid hetero-genous hyodense pancreatic lesion SUV_max_= 11.2) and few FDG avid lytic vertebral lesions were also detected. Histopathological and IHC studies enable diagnosis of synchronous lung adenocarcinoma and pancreatic ductal adeno-carcinoma with skeletal metastasis.

**Figure3:**
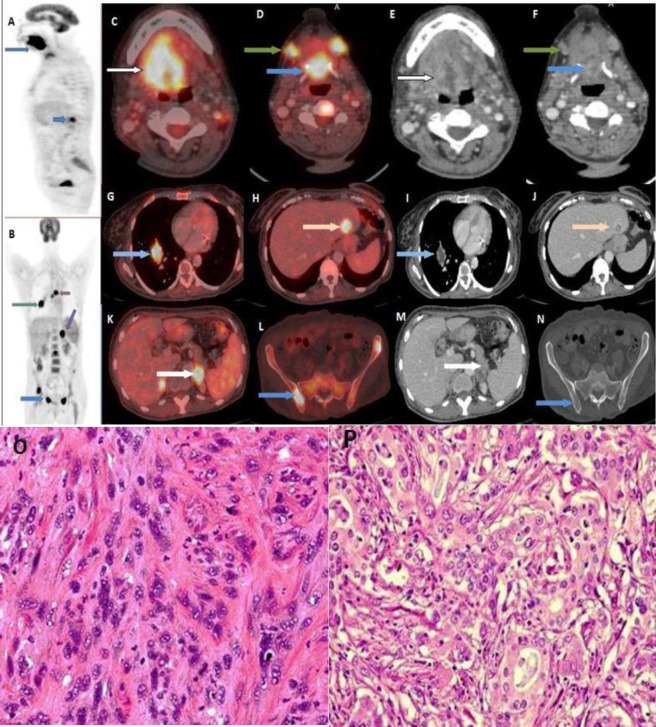
A 58 year old male diagnosed with carcinoma base of tongue extending to anterior tongue was referred for initial staging, PET maximal intensity projection (MIP) images (**A**; sagittal view) shows focally increased FDG uptake along the tongue (**blue arrow**) and anterior to the dorsal vertebra ( **short blue arrow**); (**B**. Coronal view) show focal uptake in right hemithorax (**green arrow**), dorsal vertebra ( **short arrow**), in the region of left adrenal ( **oblique arrow**) and in the pelvis ( blue arrow). Axial fused PET/CT images (**C** and **D**) and corresponding CT images (**E** and **F**) shows FDG avid lesion the tongue (**white arrow**), floor of mouth (**blue arrow**) and cervical lymph nodes (**green arrow**). Axial fused PET/CT (**G** and **H**) and corresponding CT (**I** and **J**) images show FDG avid heterogenous soft tissue lesion in the right lung lower lobe (**blue arrow**), and FDG avid hypodense lesion in left lobe of the liver (**off white arrow**). Axial fused PET/CT (**K** and **L**) and corresponding CT images (**M** and **N**) show an FDG avid ill-defined bilateral adrenal gland lesions (**white arrow**) and focal marrow based lesions in bilateral iliac bones **blue arrow**). (**0**) Biopsy of floor of mouth lesion shows non-cohesive pleomorphic cells and abnormal mitosis, permeating the connective tissues. There is no evidence of differentiation or keratinisation which lead to diagnosis of poorly differentiated SCC (H&E; 200X). (**P**) Guided biopsy of the lung lesion showed small acinar structures in desmoplastic stroma, with some of the glands contain intraluminal wispy blue mucin (H&E; 200X). Based on histopathology and IHC, diagnosis of acinar adenocarcinoma of the lung was made

 In the third case (Figure 3), a 58 year old male diagnosed with squamous cell carcinoma base of tongue with cervical lymphadenopathy was referred for initial staging. FDG/PET CT showed high grade FDG uptake in ulcerated tongue lesion (SUV_max_=14.8) and cervical lymph nodes. A high grade FDG avid heterogenous necrotic appearing lesion was also seen in floor of the mouth (SUV_max_=13.7). In addition, high grade FDG avid heterogenous soft tissue lesion in the right lung (SUV_max_=13.1), left lobe of the liver, bilateral adrenal gland lesions and focal marrow based lesions in bilateral iliac bones were also detected. Biopsy of floor of mouth lesion lead to diagnosis of poorly differentiated SCC. Guided biopsy and IHC of the lung lesion led to diagnosis of synchronous carcinomas of floor of mouth and acinar adenocarcinoma of the lung, along the metastatic lesions.

 The pathogenesis of synchronous second primary tumors can be explained by the concept of field cancerization. This concept was first introduced in 1953 by slaughter and colleagues. They reasoned that in patients with upper aerodigestive tract carcinomas, the entire epithelial surface of the upper aero-digestive tract is exposed to common carcinogens present in tobacco, alcohol and therefore has an increased risk of second primary tumors and premalignant lesions. This increased risk could be due to multiple genetic abnormalities in the whole upper aero-digestive tract (23).

 Pathogenesis of synchronous tumous ouside the aero digestive tract and below the diaphragm some patients as seen in this study can be explained by their history of exposure to certain carcinogenic risk factors. For example, one of the patients detected with synchronous stomach carcinoma had history of daily alcohol consumption and smoking over 3 decades. 

 Another patient detected with synchronous breast carcinoma was a 49 year old nulliparous woman. Other such synchronous tumors could also be explained by theory of genetic preponderance and hypothesis of polyclonal theory. According to hypothesis of polyclonal theory multiple transforming genetic events can give rise to genetically unrelated second primary tumors (24). 

 In our study, distant metastases were detected in approximately 11.7 percent patients. Distant metastases were more commonly detected among male patients and among age group of 51-70 years (Table 1). Lung was the most common site of distant metastasis, detected in 6 percent patients, followed by the skeletal metastasis in approximately 6.7 percent patients. In three percent patients metastasis was detected below the diaphragm (Table 3). 

 Distant metastases in patients with HNC have been reported in literature in the range of 4% to 25% of cases, with lungs, bones, and liver being the most frequent sites (3-6). All patients with distant metastasis had advanced stage of primary tumor corresponding to T category 3 or 4 (Table 3). In the present study distant metastasis was most common in primary carcinoma of oral cavity followed by carcinoma of hypopharynx. In a study by Gandla et al (21), 33 patients (7.03%) had distant metastasis on PET/CT scan at initial presentation and common site of distant metastasis was lung followed by bone metastasis. They described carcinoma of oral cavity (33.3%), hypopharynx

(27.2%), oropharynx (12.1%) to be more common primary lesions resulting in distant metastasis. In that study, the clinical staging of all head and neck cancer patients with distant metastasis was stage III/IV.

 Occurrence of a metastatic tumor or synchronous primary tumor in patients with the head and neck cancer carries a poorer prognosis, leads to a change in stage and treatment approach, so early identification of a metastatic or synchronous primary tumor could most benefit from an aggressive form of treatment (25). Although synchronous primary or metastatic tumors in the head and neck cancers have been described to be seen usually in the aero digestive tract, they may occur outside the aero-digestive tract and in some patients even below the diaphragm. Compared to anatomical imaging modalities like CT or MRI, whole body ^18^F-PET/CT has an advantage combining molecular and structural imaging for assessing the primary tumor, regional spread, distant metastasis and second primaries in a single sitting in patients with locally advanced head and neck cancer patients (26). However, as seen in our study, occasionally a second synchronous primary malignancy may be detected on ^18^F-FDG PET/CT scans in patients even without locally advanced HNC. Therefore whole body ^18^F-FDG PET/CT scan is a useful modality and should be routinely considered for staging and screening of patients with HNC. 

 Endoscopic procedures such as direct laryngoscopy, upper gastro intestinal endoscopy, and bronchoscopy for biopsy and subsequently histopathological and IHC confirmation of FDG PET/CT positive lesions to distinguish metastatic or synchronous tumors.

## Conclusion

 Of the distant metastasis diagnosed in 11.7% of HNC patients with TNM tumor category T3 and T4, 3% of metastasis lesions were detected below the diaphragm, which otherwise would have not been detected on conventional metastatic work up. Synchronous malignancies were diagnosed in 7% of the patients irrespective of their primary HNC stage. These results suggest that synchronous malignancy may occur irrespective of Primary HNC stage. 

 These findings demonstrate the advantage of using whole body ^18^F-FDG PET/CT for initial staging and screening of HNC patients since detection of distant metastasis or a synchronous malignancy changes the management approach in these patients. However, FDG positive lesions need careful evaluation combined with histopathological and IHC work up to differentiate disseminated metastatic lesions from synchronous primary malignancy or rarely multiple maligancies.

## Limitations of the study

The major Limitation of the study was its retrospective nature. Another limitation was a relatively small sample size which was confined to a certain geographical area. In future a study on a larger scale may be undertaken to determine the prevalence of distant metastasis and synchronous tumours in patients with head and neck cancers.

## Abbreviations used

^18^F-FDG: 18 Fluorine- Fluorodeoxyglucose

PET: Positron Emission Tomography

CT: Computed Tomography

MRI: Magnetic Resonance Imaging

PACS: Picture Archiving and Communication System

HNC: Head and Neck Cancers

SCC: Squamous Cell Carcinoma

H&E: Hematoxylin and Eosin Stain

## References

[1] Global Burden of Disease Cancer Collaboration (2017). Global, regional, and national cancer incidence, mortality, years of life lost, years lived with disability, and disability-adjusted life-years for 32 cancer groups, 1990 to 2015: a systematic analysis for the global burden of disease study. JAMA oncology.

[2] Shankar VM, Shetty RS, Salins SL, Mallya SD, Kunder MA, Bhat V (2024). IJCM_150A: A profile of Head and Neck Cancer patients attending a tertiary cancer care centre in Southern Karnataka. Indian Journal of Community Medicine.

[3] Xu GZ, Guan DJ, He ZY (2011). 18FDG-PET/CT for detecting distant metastases and second primary cancers in patients with head and neck cancer A meta-analysis. Oral oncology.

[4] Rohde M, Nielsen AL, Johansen J, Sørensen JA, Nguyen N, Diaz A (2017). Head-to-head comparison of chest x-ray/head and neck MRI, chest CT/head and neck MRI, and 18F-FDG PET/CT for detection of distant metastases and synchronous cancer in oral, pharyngeal, and laryngeal cancer. Journal of Nuclear Medicine.

[5] Park MJ, Oh JS, Roh JL, Kim JS, Lee JH, Nam SY (2017). 18F-FDG PET/CT versus contrast-enhanced CT for staging and prognostic prediction in patients with salivary gland carcinomas. Clinical Nuclear Medicine.

[6] Deurvorst SE, Hoekstra OS, Castelijns JA, Witte BI, Leemans CR, De Bree R (2018). Clinical value of 18FDG PET/CT in screening for distant metastases in head and neck squamous cell carcinoma. Clinical Otolaryngology.

[7] Mazeau-Woynar V, Cerf N (2015). Expected Survival of Cancer Patients In France: Current Situation.

[8] Coyte A, Morrison DS, McLoone P (2014). Second primary cancer risk—the impact of applying different definitions of multiple primaries: Results from a retrospective population-based cancer registry study. BMC Cancer.

[9] Jones AS, Morar P, Phillips DE, Field JK, Husband D, Helliwell TR (1995). Second primary tumors in patients with head and neck squamous cell carcinoma. Cancer.

[10] Haughey BH, Arfken CL, Gates GA, Harvey J (1992). Meta-analysis of second malignant tumors in head and neck cancer: the case for an endoscopic screening protocol. Annals of Otology, Rhinology & Laryngology.

[11] Ryu IS, Roh JL, Kim JS, Lee JH, Cho KJ, Choi SH (2016). Impact of 18F-FDG PET/CT staging on management and prognostic stratification in head and neck squamous cell carcinoma: a prospective observational study. European Journal of Cancer.

[12] Salaün PY, Abgral R, Malard O, Querellou-Lefranc S, Quere G, Wartski M (2019). Update of the recommendations of good clinical practice for the use of PET in oncology. Bulletin du Cancer.

[13] Brierley JD, Gospodarowicz MK, Wittekind C (2017). UICC TNM classification of malignant tumours.

[14] Warren S (1932). Multiple primary malignant tumors: a survey of the literature. Am J Cancer.

[15] Siddiqui MS, Chandra R, Aziz A, Suman S (2012). Epidemiology and histopathological spectrum of head and neck cancers in Bihar, a state of Eastern India. Asian Pacific journal of cancer prevention.

[16] Mishra A, Singh VP, Verma V (2009). Environmental effects on head and neck cancer in India. Journal of clinical oncology.

[17] Kekatpure V, Kuriakose MA Oral Cancer in India: Learning from different populations.

[18] Linz C, Brands RC, Hackenberg S, Hartmann S, Iring T, Hohm J (2022). [18F] FDG-PET/CT improves the detection of synchronous malignancies at primary staging of oral squamous cell carcinoma–A retrospective study. Journal of Cranio-Maxillofacial Surgery.

[19] Strobel K, Haerle SK, Stoeckli SJ, Schrank M, Soyka JD, Veit-Haibach P, Hany TF (2009). Head and neck squamous cell carcinoma (HNSCC)–detection of synchronous primaries with 18F-FDG-PET/CT. European journal of nuclear medicine and molecular imaging.

[20] Lee HS, Kim JS, Roh JL, Choi SH, Nam SY, Kim SY (2014). Clinical values for abnormal 18F-FDG uptake in the head and neck region of patients with head and neck squamous cell carcinoma. European Journal of Radiology.

[21] Gandla S, Rao V (2019). Role of PET CT in the detection of second synchronous primary tumors and distant metastasis in head and neck cancers at initial presentation. International Journal of Otorhino-laryngology and Head and Neck Surgery.

[22] Györke T, Duffek L, Bártfai K, Makó E, Karlinger K, Mester Á (2000). The role of nuclear medicine in inflammatory bowel disease A review with experiences of aspecific bowel activity using immuno-scintigraphy with 99mTc anti-granulocyte antibodies. European journal of radiology.

[23] Leclere JC, Delcroix O, Rousset J, Valette G, Robin P, Guezennec C (2020). Integration of 18-FDG PET/CT in the initial work-up to stage head and neck cancer: prognostic significance and impact on therapeutic decision making. Frontiers in Medicine.

[24] Alok A, Singh ID, Panat SR, Singh S, Kishore M (2014). Oral field cancerization: A review. Int J Dent Med Res.

[25] Graff P, Schipman B, Desandes E, Mecellem H, Toussaint B, Cortese S (2011). Management of patients with head and neck tumours presenting at diagnosis with a synchronous second cancer at another anatomic site. Clinical Oncology.

[26] Gandla S, Rao V (2019). Role of PET CT in the detection of second synchronous primary tumors and distant metastasis in head and neck cancers at initial presentation. International Journal of Otorhino-laryngology and Head and Neck Surgery.

